# Life expectancy and causes of death in Bernese mountain dogs in Switzerland

**DOI:** 10.1186/s12917-016-0782-9

**Published:** 2016-07-25

**Authors:** Michael Klopfenstein, Judith Howard, Menga Rossetti, Urs Geissbühler

**Affiliations:** 1 Vetsuisse Faculty, University of Berne, Berne, 3012 Switzerland; 2 Clinical Diagnostic Laboratory, Vetsuisse Faculty, University of Berne, Berne, 3012 Switzerland; 3 Clinical Radiology, Department of Clinical Veterinary Medicine, Vetsuisse Faculty, University of Berne, Berne, 3012 Switzerland

**Keywords:** Bernese mountain dog, Breed health, Canine, Life expectancy, Mortality, Neoplasia

## Abstract

**Background:**

New regulations by the Swiss Federal Food Safety and Veterinary Office provide for the monitoring of breed health by Swiss breeding clubs. In collaboration with the Swiss Bernese Mountain Dog Club, the purpose of this study was to investigate the causes of death in purebred dogs registered by the club and born in 2001 and 2002.

**Results:**

Of a total of 1290 Bernese mountain dogs (BMDs) born in 2001 and 2002 in Switzerland, data was collected from owners and veterinarians using a questionnaire designed for this study from 389 dogs (30.2 %). By the end of the study, 381/389 dogs (97.9 %) had died. The median life expectancy of all dogs was 8.4 years (IQR, 6.9–9.7). Female dogs had a significantly longer median survival (8.8 years; IQR, 7.1–10.3) than male dogs (7.7 years; IQR, 6.6–9.3) (*P* < 0.00). The cause of death was unknown in 89/381 dogs (23.4 %). For the remaining dogs, the most frequent causes of death were neoplasia (222/381, 58.3 %), degenerative joint disease (16/381, 4.2 %), spinal disorders (13/381, 3.4 %), renal injury (12/381, 3.1 %), and gastric or mesenteric volvulus (7/381, 1.8 %). However, large numbers of dogs were diagnosed with neoplasia without histopathologic or cytologic confirmation. Dogs with neoplasms had a shorter median survival than dogs with other disorders. The shortest median survival (6.8 years) was found for dogs with renal injury.

**Conclusions:**

Findings of this study confirm a high prevalence of neoplasia and associated low life expectancy in BMDs. The results underline a need for more widespread precise diagnostics and further research on malignant tumours in this breed to improve overall breed health.

## Background

Previous studies have demonstrated a relatively low life expectancy and high incidence of neoplasia in the Bernese mountain dog (BMD) [1–4]. Indeed, the BMD was the most short-lived breed in 1 study, with a mean life expectancy of 6.8 years [5]. More recent studies reported mean and median life expectancies of 7.1 and 7.0–8.0 years, respectively, compared with a calculated mean and median expectancy for all breeds of 10 and 11 years, respectively [1, 4, 6, 7]. Although larger breeds have been shown to have a lower average age at death than smaller breeds [1, 6, 8–10], the life expectancy of BMDs remains low when compared with breeds of similar size, such as flat coated retrievers (mean, 9.5 years) or German shepherd dogs (mean, 10.3 years) [1, 6]. In addition, some previous studies indicate a lower life expectancy of male BMDs compared to females [5]. Moreover, several studies have shown disparate longevity in dogs of all breeds based on sex and neutering, including greater longevity for neutered females compared to males or intact females [6], and shortest longevity for intact females compared to males or neutered females [10].

Several authors explain the low life expectancy in the BMD by the high incidence of neoplastic diseases [4, 7]. Indeed, previous studies reported neoplasia as the cause of death in 28.1–55.1 % of BMDs [1, 3–5] compared to 14.5–16.5 % in the overall dog population [1, 6, 10]. In particular, BMDs are predisposed to histiocytic sarcoma (HS), which is highly aggressive and associated with a very poor prognosis [4, 7, 11, 12]. Although previous studies have shown inheritance of HS in BMDs [13, 14], the exact mode of inheritance is unclear and an oligogenic transmission mode has been proposed [12]. In 1 study, examining the cause of death in a BMD population in Denmark in 2010, neoplasia (42.1 %) was the most frequently reported cause followed by old age (10.3 %), kidney disease (6.9 %), infection (5.9 %), skeletal disorders (5.2 %), heart disease (3.8 %) and behavioural disorders (3.5 %) [7].

Recent new regulations have been introduced by the Swiss Federal Food Safety and Veterinary Office (FSVO) to restrict breeding of animals with inherited defects, which are associated with pain or suffering [15]. This new administrative order also obliges breed organisations to systematically monitor their breed’s health and take measures to reduce or prevent breeding of traits that may have a negative impact on breed health. In consequence, the Swiss Bernese Mountain Dog Club launched an appeal to investigate the current state of the breed’s health in Switzerland and establish a survey system to prospectively gather epidemiologic health data. The aim of this study was to investigate life expectancy and causes of death of BMDs born in Switzerland.

## Methods

### Dog population

Data of purebred BMDs, born in Switzerland in 2001 and 2002 and registered with the Swiss Bernese Mountain Dog Club, were collected. Of a total of 1290 dogs, owners of 402 dogs (31.2 %) had participated in a previous study evaluating disease prevalence in BMDs [16], based on questionnaires completed by veterinarians and owners. Of these, data on the cause of death was available for 196 dogs. For the present study, owners of the remaining 206 dogs were contacted and invited to participate in the study to complete data on causes of death for all 402 dogs. Those agreeing were asked to have a questionnaire completed by their veterinarian at the start of the study (March 2012) and at each subsequent veterinary consultation until the end of the study (30 June 2014).

The questionnaire was modified from that used in the previous study to include only data relevant to the cause of death. The main body of the questionnaire identified the animal by name and by the 15-digit microchip number from the Animal Identity Services AG (ANIS), and served to classify diseases both by the affected organ system and the pathophysiologic disease process involved. In addition, veterinarians were asked to formulate a definitive, suspected or symptomatic diagnosis in a free-text field. Check boxes are available to identify the types of examinations conducted during consultation, treatments given and the clinical outcome. Each questionnaire was signed by both the owner and the veterinarian, confirming consent to study participation. The questionnaire was made available in German, French and English and was sent with a letter explaining the study aims to all owners of the 206 dogs in March 2012. Two months later, a reminder was sent to owners not responding to the initial contact and an attempt was made to contact those owners by telephone. A final renewed attempt to contact nonresponding owners was made in June 2013. The study was submitted for approval to the Cantonal Veterinary Services of the Canton of Berne and was determined exempt from the need for review, according to the Swiss Federal Welfare Act of December 16th 2005 and adheres. The study adheres to ARRIVE guidelines for reporting animal research.

### Encoding and reliability of diagnoses

Data collected from the completed questionnaires, the internal surveillance of the Swiss Bernese Mountain Dog Club and the ANIS Company were combined with data from the previous study [16] using a hierarchical coding system. Data regarding the cause of death or diseases identified at the time of death/euthanasia, sex and age at death were analysed. The coding system assigned diseases reported by veterinarians or owners into 1 of 12 organ systems: musculoskeletal, cardiovascular, respiratory, gastrointestinal, urogenital, integumentary, neurological, haemolymphatic, endocrine, ocular, multi-organ system, and other, whereby a multi-organ disorder was allocated if more than 1 system was identified. Disease processes were encoded into 1 of 12 classifications as inflammatory/infectious, metabolic/toxic, traumatic, neoplastic, degenerative, idiopathic, congenital, alimentary, immunologic/allergic, mechanical (such as organ displacements or obstructions), and symptomatic conditions when no clear etiologic process was identified. Data on the specific causes of death given by veterinarians were also collected.

The reliability of diagnoses was categorized as poor (cause of death reported by the owner without veterinary data or cause of death unknown), moderate (cause of death based on veterinary suspected diagnosis and clinical examination alone), high (cause of death based on veterinary suspected diagnosis, clinical examination and further supporting diagnostic examinations) or excellent (cause of death based on veterinary suspected diagnosis, clinical examination and confirmation by relevant further diagnostic examinations, whereby cytology and/or histology was necessary for the diagnosis of neoplasia). No distinction in reliability of diagnoses was made between primary-care and referral veterinarians.

### Statistical analysis

Data analysis was performed using statistical software (NCSS 9, www.ncss.com, and MedCalc Statistical Software version 15.4, MedCalc Software bvba, Ostend, Belgium; https://www.medcalc.org; 2015). Descriptive statistics were used to evaluate survival based on sex, reliability of diagnoses, organ system, disease process and specific diagnoses. Normality was evaluated using D’Agostino-Pearson tests. Kaplan-Meier survival analyses were performed to estimate and compare life expectancy based on sex, reliability of diagnoses, organ system, disease processes and specific diagnoses. Comparison of survival curves between dogs with different characteristics (such as cohort year, sex, organ system, disease process) was performed using log rank tests. Hazard ratios for the event death for specific dichotomous variables were calculated using the logrank test calculations of hazard rates derived from the cumulative hazard function of the Kaplan-Meier curves [17]. The association between specific diseases and sex was evaluated using a chi-squared test. Where a significant difference was found, the relative risk and odds ratios of male and female dogs for the specific disease were calculated, whereby the outcome variable was presence or absence of the specific disease as the cause of death and group was selected as male or female. Significance was set as *P* = 0.05 throughout.

## Results

### Population and sample characteristics

From the original target population of 402 dogs, 13 were excluded due to missing data or because owners could not be contacted or declined study participation. The target population of 389 dogs therefore represented 30.2 % of a total of 1290 BMDs born in 2001 or 2002 and registered by the Swiss Bernese Mountain Dog Club. Of these, 169/389 (43.4 %) and 220/389 (56.6 %) were born in 2001 and 2002, respectively. There were 227/389 (58.4 %) female (108 spayed, 31 intact, 88 unknown) and 162/389 male (48 intact, 53 castrated, 61 unknown) animals.

At the end of the study, 381/389 dogs were dead and 8/389 were alive (2.1 %, censored data). No distinction was made between dogs that died naturally and those euthanised. Data was available for 196 dogs from the previous study [16] and a further 130 dogs whose owners participated in the present study. In addition, data was available for 36 dogs from the Swiss Bernese Mountain Dog Club’s internal surveillance and data solely regarding the date of death of 27 dogs was available from the ANIS database.

**Fig. 1:**
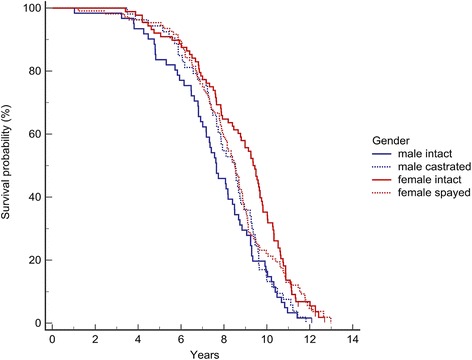
Kaplan-Meier survival curves for Bernese mountain dogs based on sex

Dogs that were dead at the end of the study, died at between 0.7 and 13.0 years of age. Dogs alive at the end of the study were between 11.5 and 12.7 years old. The mean and median survival for the entire study population was 8.25 years (95 % confidence interval (CI), 8.03–8.47) and 8.40 years (interquartile range (IQR), 6.89–9.66), respectively. No difference in life expectancy was found between dogs born in 2001 and those born in 2002 (*P* = 0.575). For all 389 dogs, females had a significantly longer life expectancy than males (*P* < 0.001) (Table 1, Fig. 1). Moreover, 7 of the 8 dogs alive at the end of the study were female. For dogs with known gonadectomy status (*n* = 310), significantly more females than males were neutered (*P* < 0.001). A significant difference in survival was found between the groups based on sex and gonadectomy status (*P* = 0.005) (Table 1, Fig. 1). No significant difference was found in survival between intact males and intact females (hazard ratio, 1.22 (95 % CI, 0.87–1.72); *P* = 0.225), between intact males and castrated males (hazard ratio, 1.18 (95 % CI, 0.82–1.71); *P* = 0.376), or between intact females and spayed females (hazard ratio, 1.19 (95 % CI, 0.90–1.58); *P* = 0.221). However, a significant difference was found between castrated males and spayed females (hazard ratio, 1.72 (95 % CI, 1.20–2.46); *P* = 0.001), castrated males having a higher hazard than spayed females (Table 1, Fig. 1).

**Table 1:** Mean and median survival of male and female Bernese mountain dogs

Factor	Sex	N	Survival (years)				P (logrank)
			Mean	95 % CI	Median	IQR	
All dogs	All	389	8.25	8.03–8.47	8.40	6.89–9.66	
	Male	162	7.80	7.49–8.12	7.73	6.62–9.29	<0.000
	Female	227	8.57	8.28–8.86	8.78	7.07–10.27	
Dogs with known gonadectomy status	All	304	8.34	8.09–8.59	8.51	6.91–9.82	
	Male intact	60	7.60	7.05–8.16	7.65	6.47–9.24	0.005
	Male castrated	53	8.18	7.67–8.69	8.53	7.23–9.45	
	Female intact	85	8.85	8.39–9.31	9.36	7.33–10.52	
	Female spayed	106	8.38	7.97–8.80	8.50	7.00–9.52	
Dogs with neoplasia	All	222	7.87	7.61–8.12	7.83	6.68–9.25	
	Male	104	7.50	7.17–7.82	7.41	6.47–8.68	<0.001
	Female	118	8.19	7.81–8.57	8.33	6.81–9.64	
Dogs with other disorders	All	70	7.78	7.23–8.78	8.00	6.74–9.29	
	Male	29	7.79	6.81–8.76	8.10	6.89–9.24	0.609
	Female	41	7.77	7.11–8.43	7.88	6.69–9.47

### Reliability of diagnoses

Of the 381 dogs that died by the end of the study, the reliability of diagnoses for the cause of death was categorized as poor in 183/381 dogs (48.0 %), moderate in 56/381 dogs (14.7 %), high in 91/381 dogs (23.9 %) and excellent in 51/381 dogs (13.4 %). The poor reliability category included 89 dogs for which the cause of death was unknown. The median life expectancy was 9.24 (IQR, 7.94–10.44), 8.17 (IQR, 6.96–9.19), 7.18 (IQR, 6.15–8.44) and 7.17 (IQR, 5.91–8.14) years for diagnoses with poor, moderate, high and excellent diagnoses, respectively.

### Causes of death

Specific causes of death recorded in more than 5 dogs were neoplasia (*n* = 222), degenerative joint disease (*n* = 16), spinal disease (*n* = 13), renal injury (*n* = 12), and gastric or mesenteric volvulus (*n* = 7).

### Organ systems

**Fig. 2:**
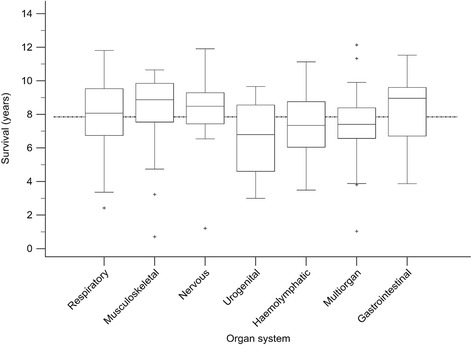
Boxplots showing survival of Bernese mountain dogs based on the organ system attributed to the cause of death

An organ system associated with the causes of death was allocated in 258 dogs and unknown in 123 dogs. The most frequently allocated organ systems were multiorgan, haemolymphatic and respiratory (Table 2). No causes of death were associated with integumentary or ocular systems. A single case of mediastinal disease was allocated to the group of other organ systems. For organ systems with at least 10 cases, a significantly shorter survival was found for multiorgan, haemolymphatic and urogenital systems compared to all other systems excluding unknown cases (Table 3, Fig. 2). A neoplastic pathologic process was involved in 60/62 and 41/51 cases of multiorgan and hemolymphatic involvement, respectively. The shortest survival was associated with disorders of the urogenital system, of which 12/14 were attributed to symptomatic conditions due to acute or chronic kidney injury.

**Table 2:** Organ systems and disease processes associated with the cause of death in Bernese mountain dogs

Organ system	Disease process									Total
	Degenerative	Immune-mediated	Inflammatory/ infectious	Metabolic/ toxic	Neoplastic	Symptomatic	Mechanical	Traumatic	Unknown	
Haemolymphatic	0	4	0	1	41	5	0	0	0	51 (13.4 %)
Nervous	0	0	0	0	5	17	0	0	0	22 (5.8 %)
Cardiovascular	0	0	0	0	1	2	0	0	0	3 (0.8 %)
Endocrine	0	0	0	1	1	0	0	0	0	2 (0.5 %)
Gastrointestinal	0	1	1	0	14	0	7	0	0	23 (6.0 %)
Musculoskeletal	16	0	0	0	21	0	0	0	0	37 (9.7 %)
Multiorgan	0	0	0	0	60	1	0	1	0	62 (16.3 %)
Other	0	0	0	0	1	0	0	0	0	1 (0.3 %)
Respiratory	0	0	1	0	42	0	0	0	0	43 (11.3 %)
Urogenital	0	0	0	0	2	12	0	0	0	14 (3.7 %)
Unknown	0	0	0	0	34	0	0	0	89	123 (32.3 %)
Total	16 (4.2 %)	5 (1.3 %)	2 (0.5 %)	2 (0.5 %)	222 (58.3 %)	37 (9.7 %)	7 (1.8 %)	1 (0.3 %)	89 (23.4 %)	381

**Table 3:** Comparison of survival based on the organ system associated with death in Bernese mountain dogs

Organ system^a^	N (%)	Survival (years)		P (logrank)^b^
		Median	IQR	
Gastrointestinal	23 (9.1)	8.96	8.96–9.60	0.162
Musculoskeletal	37 (14.7)	8.87	7.54–9.85	0.106
Nervous	22 (8.7)	8.45	7.44–9.29	0.126
Respiratory	43 (17.1)	8.07	6.75–9.54	0.154
*Overall*	*252* (*100*)	*7.85*	*6.67*–*9.12*	
Multiorgan	62 (24.6)	7.41	6.57–8.39	0.006
Haemolymphatic	51 (20.2)	7.34	6.05–8.76	0.048
Urogenital	14 (5.6)	6.76	4.61–8.56	0.033

^a^excluding cases with unknown organ systems and organ systems with less than 5 cases

^b^log rank test comparing each organ system to all others

### Disease processes and specific disorders

**Fig. 3:**
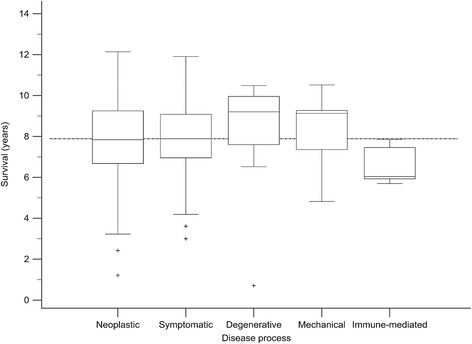
Boxplots showing survival of Bernese mountain dogs based on the disease process attributed to the cause of death

**Fig. 4:**
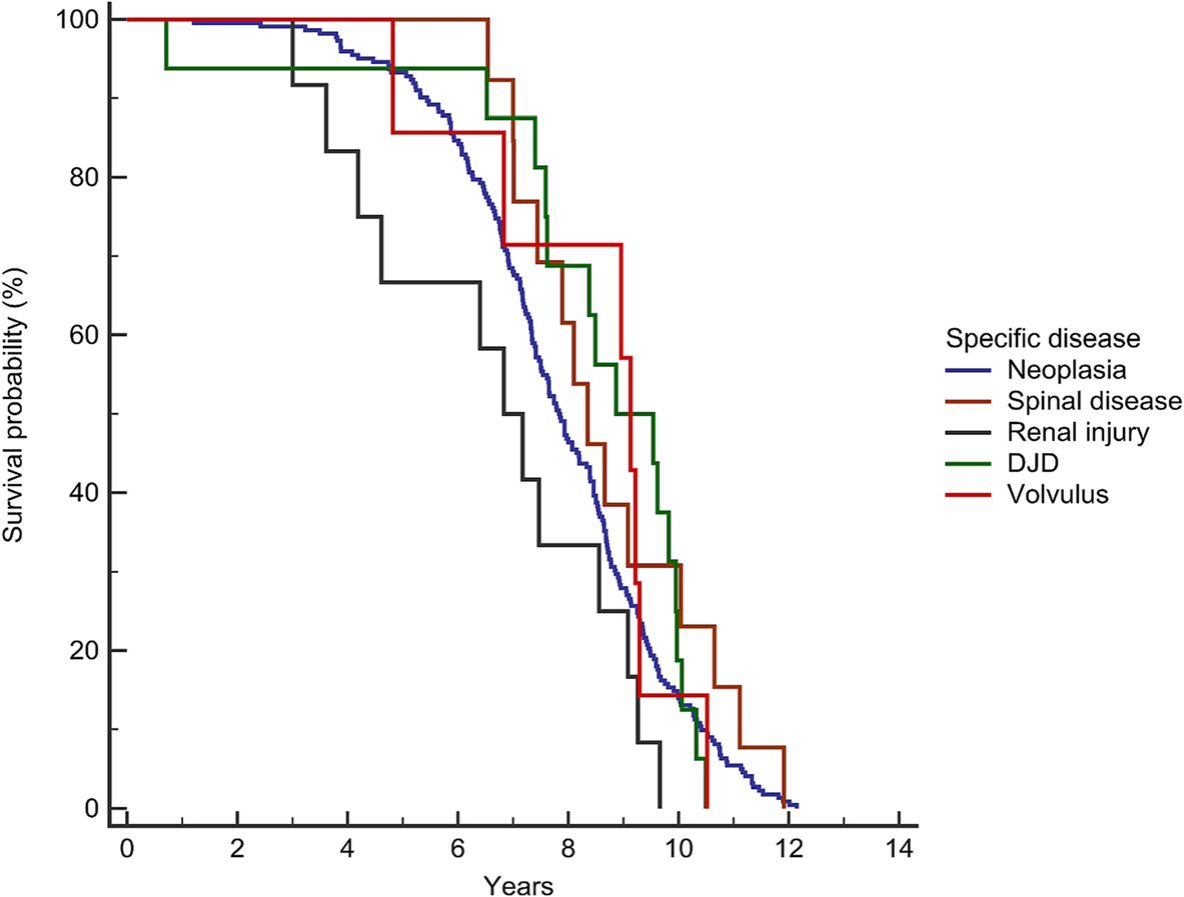
Kaplan-Meier survival curves for Bernese mountain dogs based on causes of death

No data was available for the disease process attributed to the cause of death in 89/381 dogs. For the remaining dogs, the most frequent process was neoplastic (222/381 dogs, 76.0 %) (Table 2). A further 37/381 dogs died with symptomatic conditions, most frequently associated with spinal disorders (para- or tetraplegia/paresis) (*n* = 13), acute or chronic kidney injury (*n* = 12) and anaemia (*n* = 5). All degenerative processes were attributed to degenerative joint disease and/or cruciate ligament failure. Mechanical processes were due to gastric or mesenteric volvulus. Immune-mediated processes were most frequently immune-mediated haemolytic anaemia (4 of 5 cases). Only 2 cases were attributed to inflammatory/infectious or metabolic/toxic processes, respectively, and a single case was attributed to trauma. No cause of death was attributed to idiopathic, congenital or alimentary disease processes. For disease processes with at least 5 cases, the shortest survival was associated with immune-mediated processes, followed by neoplastic processes (Table 4, Fig. 3). However, immune-mediated processes were diagnosed in only 5 cases. For specific diseases with at least 5 cases, renal injury was associated with the shortest survival, followed by neoplasia (Table 5, Fig. 4).

**Table 4:** Comparison of survival for disease processes associated with death in Bernese mountain dogs

Disease process^a^	N (%)	Survival (years)		P (logrank)^b^
		Median	IQR	
Mechanical	7 (2.4)	9.13	7.36–9.27	0.707
Degenerative	16 (5.6)	8.87	7.62–9.95	0.334
*Overall*	*287* (*100*)	*7.89*	*6.76*–*9.27*	
Symptomatic	37 (12.9)	7.89	6.96–9.08	0.898
Neoplastic	222 (77.4)	7.83	6.68–9.25	0.723
Immune-mediated	5 (1.7)	6.04	5.93–7.46	0.007

^a^excluding cases with unknown disease processes and disorders with less than 5 cases

^b^log rank test comparing each disease process to all others

**Table 5:** Comparison of survival for specific disorders associated with death in Bernese mountain dogs

Specific disorder^a^	N (%)	Survival (years)		P (logrank)^b^
		Median	IQR	
Gastric or mesenteric volvulus	7 (2.9)	9.13	7.36–9.91	0.733
Degenerative joint disease	16 (5.9)	8.87	7.59–9.95	0.352
Spinal disorder	13 (4.8)	8.35	7.33–10.19	0.206
*Overall*	*270* (*100*)	*7.93*	*6.76*–*9.29*	
Neoplasia	222 (82.2)	7.83	6.68–9.25	0.445
Renal injury (acute or chronic)	12 (4.4)	6.83	4.19–8.56	0.047

^a^excluding cases with unknown disorders and disorders with less than 5 cases

^b^log rank test comparing each disease to all others

### Neoplasia

Specific neoplasms diagnosed in the 222 dogs that died due to a neoplastic process were HS in 23/222 dogs (10.4 %), lymphoma in 15/222 dogs (6.8 %) and osteosarcoma in 11/222 dogs (5.0 %). Other tumours diagnosed on cytology or histology were sarcoma not further differentiated (*n* = 4), leukaemia (*n* = 4), fibrosarcoma (*n* = 2), haemangiosarcoma (*n* = 2), mast cell tumour (*n* = 2), transitional cell carcinoma (*n* = 1) and chondroma (*n* = 1). The remainder of neoplasms (unconfirmed on cytology or histology) were multiorgan tumours (*n* = 45), lung tumours (*n* = 29), splenic tumours (*n* = 14), liver tumours (*n* = 6), oropharyngeal tumours (*n* = 3), nervous system tumours (*n* = 2), intestinal tumours (*n* = 2), and 1 each of tumours involving the urinary bladder, heart base, hypophysis, mediastinum, nasal cavity, prostate, spine, and axial skeleton.

Histiocytic sarcomas involved the haemolymphatic, musculoskeletal, respiratory and multiorgan systems, and were diagnosed in 16 males and 7 females. Lymphoma involved the haemolymphatic and multiorgan systems, and was diagnosed in 8 males and 7 females. Osteosarcomas all involved the musculoskeletal system and were diagnosed in 6 males and 5 females.

A cause of death due to neoplasia was attributed to a significantly greater proportion of male dogs (104/161; 64.6 %) than female dogs (118/220; 53.6 %) (*P* = 0.042). Male dogs had a relative risk of neoplasia of 1.20 (95 % CI, 1.02–1.42) compared to females (*P* = 0.030), and an odds ratio of 1.58 (95 % CI, 1.04–2.40) compared to females (*P* = 0.033). Moreover, male dogs with neoplasia had a shorter survival than female dogs with neoplasia (Table 1). In addition, male dogs had a relative risk of HS of 3.13 (95 % CI, 1.32–7.42) compared to females (*P* = 0.010) and an odds ratio of 3.36 (95 % CI, 1.35–8.37) compared to females (*P* = 0.009). However, no significant difference in survival was detected between male and female dogs with HS, lymphoma or osteosarcoma. The median survival was 7.17 years (IQR, 6.50–7.84; range, 3.49 to 10.75) for dogs with HS, 7.32 years (IQR, 5.91–8.66; range 5.15 to 9.68 years) for dogs with lymphoma, and 8.74 years (IQR, 7.09–9.30; range, 4.74–10.65) for dogs with osteosarcoma.

## Discussion

The overall mean (8.25 years) and median (8.40 years) life expectancy of BMDs in the present study was higher than that reported in most studies. However, life expectancy of BMDs appears to have increased over time from 5.7 years [18] and 6.8 years [5] in early studies to between 7.0 and 8.0 years in recent years [1, 4, 7, 19]. This apparent increase in life expectancy was also noted in previous studies and has been attributed to more advanced veterinary care and an increasing social value of dogs [5, 20]. Despite this increase, the BMD remains in the group of dogs with a relatively low life expectancy compared to breed-independent values, estimated to be a mean of 10.0 years [5] or a median of 11.0 years [6, 21]. The age at death used in data analysis in the present study was based on pedigree records from the Swiss Bernese Mountain Dog Club for the date of birth and the date reported by veterinarians or owners for the date of death. The accuracy of the date of death reported could not be verified.

The low life expectancy in the BMD has been attributed to a high prevalence of neoplasia, reported to be between 26.1 % and 55.1 % [1, 3–5, 18, 19, 22], compared to other purebred and general dog populations, in which tumour prevalence is estimated between 14.5 % and 27 % [1, 6, 21]. Data in the present study corroborates these findings with death due to neoplasia in 58.3 % of the target population. This was somewhat higher than previously reported. However, the reliability of diagnoses in the present study was highly variable, and most cases with a diagnosis of neoplasia were not confirmed on cytology or histology. The true incidence of neoplastic disorders is therefore likely to be somewhat lower or higher than that found in the present study.

Female BMDs in the present study had a higher life expectancy than males, corroborating findings of a previous study in which female BMDs were reported to live about 1 year longer than males [5]. Although a higher hazard ratio was found for intact males compared to intact and spayed females, and castrated males compared to intact females, this was not detected for castrated males compared to spayed females. However, a large proportion of dogs, for which the gonadectomy status was unknown, was excluded from this analysis. Moreover, the time at which gonadectomy took place was not taken into consideration. The effect, if any, of long-term presence or absence of sex hormones on survival is therefore unclear. Possible explanations for longer survival in females include a generally higher life expectancy in female dogs, as well as the higher risk of neoplasia in males and the associated lower survival in males in the present study. This finding contradicts findings of a previous study in which no difference in risk of neoplasia was found between males and females [4]. Moreover, it is in contrast to findings of different cancer registries, which reveal a predisposition for all neoplasms in female dogs [2, 5, 23]. However, this female predisposition is largely due to mammary cancer, which was not found in any of the dogs in the present study and was found to have a low prevalence in BMDs in a previous study [5]. This finding suggests that, although BMDs may be predisposed to some neoplasms, they may have a diminished risk for certain other types of neoplastic disorders or simply not live long enough for neoplasms associated with high age.

The most frequently diagnosed neoplasia in the present study was HS (10.4 % of all neoplasms). This was similar to previous investigations in Dutch and Norwegian BMD populations, in which HS accounted for 15.3 % [4] and 10.7 % [7], respectively. In a study of BMDs presented to a German university hospital, HS accounted for 8.2 % of histologically-confirmed tumours but up to 15.7 % when including tumours not histologically confirmed but highly suspected based on typical lesion distribution [22]. As the majority of tumours diagnosed in the present study lacked histologic confirmation and many dogs died without any diagnosis, the true prevalence in this population is unclear.

Of dogs with confirmed HS, the median survival of 7.2 years was somewhat higher than the previously reported 6.3 years [4]. Previous studies reported a wide range of ages of BMDs diagnosed with HS with one report of dogs aged between 10 months and 14.7 years with the majority between 5 and 8 years old [22]. Findings in the present study were similar with nearly half of the dogs dying between 7 and 8 years old with a range between 3.5 and 10.8 years old. The higher relative risk of male dogs with HS in the present study does not corroborate findings of previous recent studies in which females were reported with a higher prevalence or no difference in sex was found [11, 12, 22]. The reason for this apparent discrepancy is unclear but the large numbers of dogs in the present study for which no cause of death was available introduces a large uncertainty with regards to disease prevalence and sex distributions. Lymphoma was the second most commonly identified cancer, corroborating findings in previous studies [22].

With regards to non-neoplastic disease processes, dogs with symptomatic conditions included 13 dogs that were euthanised or died with paraparesis, paraplegia or tetraparesis. The median life expectancy of these dogs was 8.4 years, suggesting that at least some of these dogs may have suffered from degenerative myelopathy, previously described in older dogs of this breed [24]. However, this cannot be confirmed as post-mortem histopathology was not performed in any of these dogs. The BMDs with urogenital disorders had the shortest life span and most were attributed symptomatically to chronic or acute kidney injury. The extent to which any of these dogs was affected by hereditary membranoproliferative glomerulopathy in this breed is unclear [25, 26]. All in all, the 3.6 % of deaths due to kidney injury is somewhat lower (6.9 %) than in a previous report [7]. Five dogs suffered from immune-mediated diseases, of which 4 were diagnosed with immune-mediated haemolytic anaemia or Evans syndrome, resulting in a particularly low life expectancy in this disease process group. However, the number of dogs was very low and the degree to which an underlying neoplastic process was ruled out is not known.

In contrast to 1 previous study [7], cardiovascular diseases, behavioural problems, and infectious diseases occurred extremely rarely or not at all in our limited sample population. However, behavioural problems as cause of death (euthanasia) might not have been reported, as euthanasia for this reason is considered somewhat taboo in our society. In addition, a high vaccination coverage for common infectious diseases in Switzerland and good access to veterinary care might diminish the rate of lethal infectious diseases. Moreover, treatment of disorders such as degenerative joint diseases may significantly diminish their prevalence as a cause of death even though they may be widespread chronic disorders within the population. As the present study only evaluated diseases associated with death, the data cannot therefore be interpreted as general prevalence data.

The reason for a short lifespan in the BMD is likely multifactorial but significant factors include the high prevalence of tumours, as only few other breeds, including flat-coated retrievers and boxers, exhibit a comparable high prevalence [2–4]. Furthermore, BMDs with neoplasia died with a median age of 1.2 years younger than those that died of causes other than neoplasia in the present study. The shorter life span for dogs of larger body size is an additional factor [1, 8–10, 27–29].

In the present study, information regarding the cause of death that was purely based on information acquired from dog owners was considered of low diagnostic reliability. Interestingly, dogs with higher longevity were more frequently classified in the lowest quality of diagnoses. In contrast, dogs that died at an early age were classified in the highest categories. This observation may suggest that dog owners and veterinarians are more willing to invest time and money in the work-up and diagnosis of diseases in dogs presenting at a younger age. However, it is also possible that far more detailed data was transmitted by veterinarians when dogs were euthanized at a younger age.

To the authors’ knowledge, this is the first study to investigate only purebred BMDs with pedigree born in a single country and representing a large proportion of all dogs born within the same period. As the vast majority of dogs (all but 8) in the present study had died by the end of the study, bias due to right-censored data was minimal in the present study. Major limitations of the study include a relatively large proportion of owners who chose not to participate or could not be located, creating possible responder bias. In addition, slightly over 20 % of dogs died or were euthanised without any specific diagnosis, and the diagnosis of neoplasia was mostly made without histologic confirmation. Moreover, a large proportion of diagnoses were of poor or moderate reliability. Possible reasons include unwillingness of owners to confirm diagnoses in cases of presumed poor prognosis, and unwillingness to perform post-mortem examinations in cases in which no specific antemortem diagnosis was evident. For owners unwilling to perform necropsy for emotional and/or spiritual reasons, minimally invasive digital imaging, such as X-Ray, Ultrasound, CT and MRI in combination with fine needle aspiration and needle biopsy, may constitute a promising tool to improve post-mortem diagnoses [30]. In addition, overdiagnosis of neoplasia by veterinarians because of a perceived high risk of tumours in BMDs may have artificially inflated prevalence of neoplasia in this study. At the same time, some animals with unknown cases of death or those dying with only symptomatic diagnoses may indeed have had undiscovered neoplasia, leading to underestimation of its true prevalence.

Besides analyses on the causes of death, the collection of health histories from birth to death, as well as clinical and genetic material for analyses of specific diseases may be valuable to more precisely assess breed health. Further studies, prospectively collecting health data from defined health populations or cohorts may enable breeding clubs to more accurately assess breed health.

## Conclusions

This study confirms the high prevalence of neoplasia in BMDs. Although the prevalence may be overestimated due to the lacking histological or cytological confirmation, neoplasia is an important factor for the low life expectancy in BMDs.

The diagnostic accuracy leaves much to be desired and further research on malignant processes with a high diagnostic standard is necessary to improve breed health.

## Abbreviations

ANIS, Animal Identity Services AG; BMD, Bernese mountain dog; HS, Histiocytic sarcoma

## References

[1] Proschowsky HF, Rugbjerg H, Ersbøll AK (2003). Mortality of purebred and mixed-breed dogs in Denmark. Prev Vet Med.

[2] Brønden LB, Nielsen SS, Toft N, Kristensen AT (2010). Data from the Danish veterinary cancer registry on the occurrence and distribution of neoplasms in dogs in Denmark. Vet Rec.

[3] Fleming JM, Creevy KE, Promislow DEL: Mortality in North American dogs from 1984 to (2004). An Investigation into age-, size-, and breed-related causes of death. J Vet Intern Med.

[4] Erich S, Rutteman G, Teske E (2013). Causes of death and the impact of histiocytic sarcoma on the life expectancy of the Dutch population of Bernese mountain dogs and flat-coated retrievers. Vet J.

[5] Eichelberg H, Seine R. Lebenserwartung und Todesursachen bei Hunden I. Zur Situation bei Mischlingen und verschiedenen Rassehunden. Berliner und Münchner Tierärtzliche Wochenschrift. 1996;109:292–303. 9005839

[6] Michell AR (1999). Longevity of British breeds of dog and its relationships with sex, size, cardiovascular variables and disease. Vet Rec.

[7] Nielsen L, Andreasen SN, Andersen SD, Kristensen AT (2010). Malignant histiocytosis and other causes of death in Bernese mountain dogs in Denmark. Vet Rec.

[8] Patronek GJ, Waters DJ, Glickman LT (1997). Comparative longevity of pet dogs and humans: implications for gerontology research. J Gerontol.

[9] Greer KA, Canterberry SC, Murphy KE (2007). Statistical analysis regarding the effects of height and weight on life span of the domestic dog. Res Vet Sci.

[10] O’Neill DG, Church DB, McGreevy PD, Thomson PC, Brodbelt DC (2013). Longevity and mortality of owned dogs in England. Vet J.

[11] Affolter VK, Moore PF (2002). Localized and disseminated histiocytic sarcoma of dendritic cell origin in dogs. Vet Pathol.

[12] Abadie J, Hédan B, Cadieu E, De Brito C, Devauchelle P, Bourgain C, Parker HG, Vaysse A, Margaritte-Jeannin P, Galibert F, Ostrander E, André C (2009). Epidemiology, pathology, and genetics of histiocytic sarcoma in the Bernese mountain dog breed. J Hered.

[13] Padgett G, Madewell BR, Keller ET, Jodar L, Packard M (1995). Inheritance of histiocytosis in Bernese mountain dogs. J Small Anim Pract.

[14] Voegeli E, Welle M, Hauser B, Dolf G, Flückiger M (2006). Das histiozytäre Sarkom beim Berner Sennenhund in der Schweiz: Eine retrospektive Studie über seine genetische Prädisposition. Schweizerisches Arch für Tierheilkd.

[15] Verordnung des BLV über den Tierschutz beim Züchten. In *Swiss Fed Food Saf Vet Off*; 2014:1–11. https://www.admin.ch/opc/search/?lang=de&language%5B%5D=de&product%5B%5D=oc&text=Tierschutz+beim+Z%C3%BCchten&lang=de.

[16] Rossetti M, Doherr M, Geissbühler U (2011). Morbidity and mortality in Bernese mountain dogs with pedigree born in Switzerland in 2001 and 2002. Dr Diss Univ Berne.

[17] Bewick V, Cheek L, Ball J (2004). Statistics review 12: survival analysis. Crit Care.

[18] Berdal WP, Moe L, Glattre E (1994). Demographic characteristics of Bernese mountain dogs in Norway. Kenya Vet.

[19] Report from the Kennel Club / British Small Animal Veterinary Association Scientific Committee: summary results of the purebred dog health survey for Bernese mountain dogs [http://www.thekennelclub.org.uk/vets-researchers/purebred-dog-health-survey-results/]

[20] Bonnett BN, Egenvall A (2010). Age Patterns of Disease and Death in Insured Swedish Dogs, Cats and Horses. J Comp Pathol.

[21] Report from the Kennel Club / British Small Animal Veterinary Association Scientific Committee Summary results of the Purebred Dog Health Survey for all breeds [http://www.thekennelclub.org.uk/vets-researchers/purebred-dog-health-survey-results/]

[22] Coenen C (2009). Untersuchungen zur Häufigkeit und klinischem Erscheinungsbild des histiozytären Sarkoms beim Berner Sennenhund unter besonderer Berücksichtigung zytologischer Knochenmarkanalysen.

[23] Merlo DF, Rossi L, Pellegrino C, Ceppi M, Cardellino U, Capurro C, Ratto A, Sambucco PL, Sestito V, Tanara G, Bocchini V (2008). Cancer incidence in pet dogs: findings of the Animal Tumor Registry of Genoa, Italy. J Vet Intern Med.

[24] Zeng R, Coates JR, Johnson GC, Hansen L, Awano T, Kolicheski A, Ivansson E, Perloski M, Lindblad-Toh K, O’Brien DP, Guo J, Katz ML, Johnson GS (2014). Breed distribution of SOD1 alleles previously associated with canine degenerative myelopathy. J Vet Intern Med.

[25] Minkus G, Breuer W, Wanke R, Reusch C, Leuterer G, Brem G, Hermanns W (1994). Familial nephropathy in Bernese mountain dogs. Vet Pathol.

[26] Reusch C, Hoerauf A, Lechner J, Kirsch M, Leuterer G, Minkus G, Brem G (1994). A new familial glomerulonephropathy in Bernese mountain dogs. Vet Rec.

[27] Li Y, Deeb B, Pendergrass W, Wolf N (1996). Cellular proliferative capacity and life span in small and large dogs. J Gerontol Ser A Biol Sci Med Sci.

[28] Galis F, van der Sluijs I, Van Dooren TJM, Metz JAJ, Nussbaumer M (2007). Do large dogs die young?. J Exp Zool (Mol Dev Evol).

[29] Kraus C, Pavard S, Promislow DEL (2013). The size–life Span trade-off decomposed: Why large dogs die young. Am Nat.

[30] Hostettler FC, Wiener DJ, Welle MM, Posthaus H, Geissbühler U (2015). Post mortem computed tomography and core needle biopsy in comparison to autopsy in eleven Bernese mountain dogs with histiocytic sarcoma. BMC Vet Res.

